# Economic Evaluation of a Digital Psychobehavioral Intervention for Chronic Inflammatory Skin Disease (MindMySkin): Protocol for a Trial-Based Cost-Effectiveness Analysis

**DOI:** 10.2196/95215

**Published:** 2026-09-29

**Authors:** Ellie Choi, Sin Hui Neo, Phillip Phan, Stavros Petrou

**Affiliations:** 1 Division of Dermatology, Department of Medicine, National University Hospital, Singapore, Singapore; 2 Nuffield Department of Primary Care Health Services, Health Economics and Policy Evaluation, University of Oxford, Radcliffe Primary Care Building, Radcliffe Observatory Quarter Woodstock Rd, Oxfordshire, England, OX2 6GG, United Kingdom, 44 7346879730; 3 Centre for Research in Health Systems Performance, Yong Loo Lin School of Medicine, National University of Singapore, Singapore, Singapore; 4 Carey Business School, Johns Hopkins University, Baltimore, MD, United States

**Keywords:** economic evaluation, cost-effectiveness analysis, health technology assessment, digital health, mobile health, mHealth, eczema, psoriasis, dermatology, psychological intervention, mental health

## Abstract

**Background:**

Digital mental health apps are increasingly available and used, yet the large majority lack clinical and economic evidence, limiting reimbursement support and the identification of high-value interventions. MindMySkin is a digital psychobehavioral mobile intervention co-designed with patients and guided by behavioral change frameworks to address the psychobehavioral determinants of symptom burden in chronic inflammatory skin diseases. The app comprises 74 modules targeting illness coherence, symptom management, and emotional and functional impairment and is currently being tested in a double-blind randomized controlled trial.

**Objective:**

This protocol prespecifies an economic evaluation plan conducted as a secondary analysis alongside the trial to assess the cost-effectiveness of MindMySkin within Singapore’s health care system.

**Methods:**

The parent trial recruits English-speaking participants aged 16 years or older with psoriasis, eczema, or chronic urticaria across 3 sites in Singapore (target N=690; 2:1 allocation, 32-week follow-up). The active comparator is Healthy365, a general wellness app managed by the Singapore Health Promotion Board. The trial-based economic evaluation will adopt a health care system perspective as the primary analytic viewpoint. Resource costs will include intervention deployment and dermatology-related health care use. Indirect medical costs and work productivity losses will additionally be included in a sensitivity analysis conducted from a societal perspective. A cost-effectiveness analysis will use the Dermatology Life Quality Index, a dermatology-specific measure of health-related quality of life, while a cost-utility analysis will derive health utilities from the EQ-5D-5L to enable estimation of quality-adjusted life years. Incremental cost-effectiveness ratios and net monetary benefit will be estimated using intention-to-treat analyses with regression adjustment. Uncertainty will be explored using bootstrapping, deterministic sensitivity analyses, subgroup analyses, and scenario modeling. A Markov model–based extrapolation over 5 years will be undertaken if within-trial results suggest a high likelihood of cost-effectiveness.

**Results:**

Study funding is provided by the National Medical Research Council Clinical-Scientist Individual Research Grant New Investigator Grant (CS-IRG NIG; funded on June 5, 2024), and ethics approval has been obtained (2022/00751). Recruitment commenced in February 2026, and as of June 2026, a total of 46 participants have been enrolled. Economic data collection will be conducted concurrently with the parent trial, and the economic analysis is expected to commence following trial completion, which is anticipated in 2028.

**Conclusions:**

By embedding a health economic evaluation within a randomized controlled design and incorporating condition-sensitive outcome measures, this study advances methodological standards for evaluating scalable psychobehavioral interventions. Demonstrating value through disability reduction rather than survival gain may strengthen the legitimacy of low-intensity digital therapeutics within health technology assessment frameworks and inform implementation decisions in resource-constrained health systems.

## Introduction

Digital mental health interventions have expanded rapidly over the past decade, driven by their increased acceptability, accessibility, and scalability. Global market analyses project a compound annual growth rate of approximately 20%, with more than 20,000 mental health apps currently available across consumer app store platforms [1,2]. Unlike pharmaceuticals and medical devices, digital mental health apps generally do not require demonstration of clinical efficacy or cost-effectiveness prior to deployment, and more than 90% are not supported by original research [3]. Consequently, many enter the ecosystem with limited evidence of benefit, receiving limited clinician endorsement and reimbursement support while potentially exposing patients to low-value interventions.

Conceptually, self-guided mental health apps can represent an intermediate care delivery pathway between therapist-guided treatment and no psychological care. Recent studies, including a meta-analysis of 176 randomized controlled trials (RCTs), found that these apps produced modest reductions in symptoms of depression (*g*=0.28; number needed to treat=11.5) and generalized anxiety (*g*=0.26; number needed to treat=12.4) [4-7]. While self-guided apps may impose greater system-level costs than not providing any psychological care, they remain considerably cheaper to deploy than therapist-guided treatments [8-10]. This raises the central health economic question of whether the clinical benefits of digital mental health apps justify their adoption and deployment costs when compared with a counterfactual scenario involving no psychological intervention.

This economic evaluation protocol of MindMySkin, a digital mental health app for dermatological disease, extends this evidence base in 2 important ways. First, it evaluates the intervention in the context of a defined chronic medical condition rather than in a general population or in a sample selected primarily for anxiety or depression. This distinction is clinically important because psychological morbidity is often shaped by disease-specific mechanisms. In dermatology, it includes visible lesions, pruritus, discomfort, stigma, treatment burden, sleep disruption, and restrictions in social and occupational functioning. Second, the protocol emphasizes functional disability and health-related quality of life (HRQOL) as primary treatment-relevant outcomes, rather than evaluating depressive and anxiety symptoms as stand-alone end points. This approach is important because depression and anxiety are clinically important not only as symptom clusters but also because of their effects on daily functioning, disability, social participation, work productivity, and HRQOL. In chronic inflammatory skin diseases, HRQOL impairment is strongly determined by psychological and behavioral factors, including illness perceptions, negative skin-directed behaviors, and maladaptive coping, more so than objective disease severity itself [11-14]. Furthermore, symptomatic improvement does not necessarily indicate full functional recovery [15], and HRQOL assessment is recognized as an important complement to symptom-based measures.

MindMySkin was developed as an adjunctive intervention to address psychobehavioral factors that are often unaddressed in existing care pathways, which tend to focus primarily on biomedical treatments and therapies [13,16,17]. In brief, the development was undertaken by a multidisciplinary team comprising patients, psychologists, and psychodermatologists following the UK Medical Research Council Framework for Complex Interventions [18]. Development was guided by a theory of change model [19,20], which systematically mapped identified patient needs to psychotherapeutic techniques and intended outcomes. The selection of intervention modules and behavior change techniques was guided by a literature review; expert opinion; and the capability, opportunity, motivation–behavior model and behavior change wheel [21]. These ultimately comprised 74 modules covering knowledge delivery, cognitive behavioral therapy, habit reversal training, and mindfulness training, among others. The intervention was co-designed with patients and iteratively tested. The modules are delivered through structured educational modules, guided exercises, journaling, and on-demand support tools. A full account of the development process and theoretical framework is separately published [17]. In the context of constrained health care resources, clinical benefit must be assessed alongside costs to determine whether MindMySkin represents value for money. This economic evaluation therefore aims to determine the cost-effectiveness of MindMySkin compared with usual care, to inform prioritization, adoption, and implementation decisions within broader clinical pathways.

## Methods

### Dataset

The evidence base for this economic evaluation comprises a multicenter, double-blind RCT of MindMySkin, currently being conducted across 3 outpatient dermatological centers in Singapore, with a target recruitment of 690 participants with psoriasis, eczema, or chronic urticaria [22]. Participants are randomized to MindMySkin or the Healthy365 app, which serves as an active control comparator. Healthy365 is a general wellness app managed by the Singapore Health Promotion Board, providing health tracking, fitness challenges, and general lifestyle recommendations without specifically targeting skin disease or psychodermatological outcomes. The use of an active control preserves blinding and helps differentiate intervention-specific effects from broader nonspecific effects associated with app use and health engagement. This is particularly important for the economic evaluation, as attributing cost-effectiveness to MindMySkin’s targeted psychobehavioral content, rather than to app engagement per se, strengthens the validity of the incremental analysis and the relevance of findings for implementation decisions. For the economic evaluation, effectiveness is assessed using the Dermatology Life Quality Index (DLQI) [23], while health utility is measured using the EQ-5D-5L [24], enabling derivation of quality-adjusted life years (QALYs) across the 32-week study period.

### Economic Evaluation Framework

This study conducts a cost-utility and cost-effectiveness evaluation in accordance with the Expert Delphi Consensus for Health Economics Analysis Plans for Trial-Based Economic Evaluations [25] and the Consensus on Health Economic Criteria checklist [26].

### Study Population and Setting

This double-blind RCT will recruit English-speaking participants aged 16 years or older with psoriasis, eczema, or chronic urticaria across 3 study sites in Singapore. The restriction to English-speaking participants reflects the current availability of MindMySkin in English only. The age cutoff of 16 years or older is intended to ensure participants have sufficient cognitive maturity to self-manage a digital psychobehavioral intervention. Participants will be randomized in a 2:1 ratio to MindMySkin or an active control (Healthy365 [27]) and followed up for 32 weeks. The full protocol has been separately published [22].

### Study Perspective

All study sites are health care institutions operating within Singapore’s publicly funded health system, in which services are financed through a combination of patient payments, patient subsidies, and institution-side government subvention. The primary base-case analysis will adopt a Singapore health care system perspective, as recommended by the Agency for Care Effectiveness Singapore [28], whereby costs to the system as a whole will be considered. A complementary analysis will be conducted from the societal perspective, including patient out-of-pocket costs and productivity costs.

The cost analysis aims to inform the cost-effectiveness associated with the deployment and maintenance of MindMySkin as a scalable service beyond the trial setting. Intervention development costs and research costs will be collected separately but excluded from the primary analysis.

### Economic Hypotheses and Assumptions

MindMySkin is hypothesized to improve HRQOL through reductions in symptom burden and disability, resulting in changes in health state utility without effects on mortality or life expectancy. The economic evaluation thus assumes no life years gained, with all changes in QALYs attributable to changes in HRQOL outcomes over time. As a supportive, nonsubstitutive intervention, MindMySkin is not expected to materially alter patterns of health care use. Productivity improvements in work, school, and daily activity are anticipated in the intervention arm as downstream effects of reduced symptom burden and improved coping. These effects will be examined in secondary analyses from the societal perspective.

### Intervention and Comparator

MindMySkin [17] is self-administered, delivered via a mobile app, and designed to complement routine dermatological care. The content is self-paced, does not require clinician involvement, and allows users to engage at their discretion, thereby reflecting real-world use beyond the trial setting.

The comparator is the current standard of care, operationalized as an active digital control using the Healthy365 app, a nationally funded general wellness app that does not include dermatology-specific or psychobehavioral therapeutic content. Healthy365 is an existing publicly funded program with fixed sunk costs independent of this study. Therefore, no app-related costs are attributed to the control arm. In a similar vein, structured psychobehavioral interventions are not part of routine dermatological care in Singapore, and the comparator is assumed to incur no additional intervention-related costs. Participants in both arms will continue to receive standard medical management, including clinic visits, investigations, and pharmacological treatments when clinically indicated.

### Time Horizon

The within-trial economic evaluation adopts a 32-week time horizon, corresponding to the final follow-up of the RCT. This horizon captures the full period over which costs and health outcomes are prospectively observed and is appropriate for assessing short- to medium-term differences in health care use, HRQOL, and symptom burden attributable to the intervention. For planning and interpretive purposes, a longer-term horizon (up to 5 years) is considered in scenario and model-based extrapolation analyses to explore the potential implications of sustained use and scale-up. This is explicitly exploratory and not part of the base-case trial-based analysis.

### Measurement and Valuation of Costs

#### Intervention Development Cost

Development of the intervention took place between 2022 and 2025, with costs prospectively documented to enhance transparency regarding upfront investments. Manpower inputs were recorded using a combination of prospectively completed activity log sheets supplemented by retrospective review of project documentation. Unit costs, including salary valuations, were obtained from institutional finance schedules, inclusive of employer on-costs, and supplemented with national cost reference data where appropriate. Where institutional overheads were applied, these reflected standard indirect cost rates used by the hosting health care institution.

Using a bottom-up microcosting approach, we will sum personnel, content development, design, usability testing, and pilot implementation costs across all stages of the MindMySkin intervention. Research costs associated with trial conduct and outcome evaluation would not be incurred under routine deployment and therefore will be excluded. Costs will be calculated in Singapore dollars for the year 2025, in which the app development was completed.

Although these costs are excluded from the base-case economic evaluation, the nonrecurring, one-off development costs provide important contextual information for stakeholders, including health care systems, institutions, and public agencies considering similar in-house development of digital psychobehavioral programs. We will therefore document these costs to support planning, replication, adaptation, and potential scale-up across alternative settings.

#### Intervention Deployment Cost

Costs related to deployment will be categorized into fixed costs, defined as one-off or capacity-enabling investments required to support delivery of the intervention, and variable costs that increase with the number of users (Textbox 1).

Textbox 1. **Fixed and variable deployment costs.**

**Fixed deployment costs**

- Dermatological content updates and periodic redesign
- Implementation-related training for clinic staff
- Legal and regulatory compliance costs, including governance and audit
- Core technology infrastructure, baseline hosting environment, and platform-level support

**Variable (incremental deployment cost)**

- Administrative and operational support for onboarding and patient education
- User-level technical support and troubleshooting

Fixed deployment costs include required dermatological content updates and app redesign, technology infrastructure and hosting, technical maintenance and support, legal and regulatory requirements necessary for service operation (including quality assurance, audit, and governance), and implementation-related training and materials.

Variable (incremental) deployment costs include per-participant licensing or user fees (where applicable) and administrative and operational support related to user onboarding and troubleshooting. These costs increase with the scale of implementation.

All deployment costs will be adjusted to the 32-week study period.

An incremental costing approach is adopted, wherein only the resources that differ between the intervention and control arms are included. In the base-case analysis, current standard care does not include a psychobehavioral component and therefore incurs no additional intervention-related costs; accordingly, it is costed as zero. There are no capital costs that require annuitization.

### Determination of Potential User Base

The primary estimate of the potential user base for the intervention, over which fixed intervention costs are allocated, is derived using a site-based approach reflecting uptake within participating clinical settings. Each participating site will provide standardized information for a reference year (eg, 2025), including the annual number of unique dermatology outpatients and the disease distribution for atopic dermatitis/eczema, psoriasis, and chronic urticaria. Where diagnosis-specific counts are unavailable, percentage estimates will be derived from a small sample of representative clinic sessions.

Each site will estimate the proportion of patients with sufficient English proficiency, educational level, and digital literacy to engage with the intervention. To ensure consistency across sites, sampling of a fixed number of clinic sessions (eg, 5) will be used where applicable. In addition, each site will estimate the proportion of suitable patients who would realistically use the app if offered as part of routine care, informed by direct patient queries where feasible or, alternatively, by clinician judgment. This approach is anchored to patients attending the recruiting sites only and does not assume broader population-level uptake.

A second estimate of the potential user base will be derived using a platform-anchored approach to define a lower bound of potential uptake. This approach will be based on Intellect’s (the health technology platform hosting MindMySkin’s content) existing active user base and estimated prevalence of atopic dermatitis or eczema, psoriasis, and chronic urticaria. This approach assumes that only individuals who are already active users of the Intellect platform and have one of these dermatological conditions would adopt the skin-specific modules. The conservative estimation of true uptake ensures that economic benefits are more likely to be underestimated than overestimated.

### Broader (Downstream) Health Care Use and Cost

Broader health care resource use will be captured using a resource use questionnaire adapted from the Core Items for a Standardized Resource Use Measure, which was developed through an expert Delphi consensus process [29]. Resource categories for the primary analysis will include domains most plausibly influenced by MindMySkin, including outpatient dermatology clinic visits, other relevant dermatological visits, emergency department attendances, inpatient admissions related to skin disease, and dermatology-related treatments and medications. Certain items and domains that were judged to be less relevant to the target ambulatory outpatient dermatology population (eg, hospice and rehabilitation care arrangements) were removed to reduce response burden, misattribution, and noise introduced by random variation in all-cause health care use over the study period, improving internal validity and avoiding inappropriate cost inflation. The adapted questionnaire covering resource categories in Table 1 is being administered at weeks 16 and 32 using a 4-month recall period.

**Table 1.** Health care and wider resource categories and valuation methods^a^.

Resource	Valuation
Health care system perspective	
Outpatient dermatology clinician visit	Natural units×unit cost
Outpatient dermatology nonclinician visit	Natural units×unit cost
Outpatient visit with a general practitioner or primary care	Natural units×unit cost
Emergency or urgent care visit for a skin-related condition	Natural units×unit cost
Inpatient admission for a skin-related condition (primary or secondary), including reason for and duration of admission	Natural units×bed-days×unit cost
Dermatology-related ancillary costs	
Laboratory tests and diagnostics	Amount spent (self-reported, supplemented by institutional data)
Medications	Amount spent (self-reported, supplemented by institutional data)
Other treatments (eg, phototherapy and injections)	Amount spent (self-reported, supplemented by institutional data)
Societal perspective	
Skin-related out-of-pocket expenditures	
Nonprescriptive medications	Amount spent (self-reported)
Complementary or alternative treatments	Amount spent (self-reported)
Transportation and travel-related costs	Amount spent (self-reported)
Work, school, and other productivity losses	Degree of absenteeism and presenteeism monetized using the national average wage
Estimated time spent managing the skin condition	Presented descriptively as hours

a Resource use is self-reported by participants at weeks 16 and 32, with a 4-month recall period and valued using institutional financial data.

Resource use will be self-reported in natural units, reflecting the actual volume of services consumed by each participant during the study period, and valued using institution-specific billing data and unit costs. In the absence of unit cost data, patient charges before subsidies will be used as a proxy for the cost of service provision. This is consistent with the practice in Singapore’s public health care institutions, which typically charge for services on a cost-recovery basis [30,31], and is an approach used in prior Singapore studies [32,33]. Additional studies further support the use of charges where the primary interest is the estimation of incremental cost differences between groups [34]. Medication costs will be valued using retail and institutional drug formulary prices obtained from institutional pharmacy schedules and the national drug information database.

### Societal Costs

A broader societal perspective will be considered by including patients’ out-of-pocket expenditures, costs associated with productivity losses, and monetized time costs (Table 1). Out-of-pocket expenditures and nonmedical costs (including complementary or alternative treatments and transportation) will be captured using the same self-administered resource use questionnaire, administered at weeks 16 and 32 alongside the primary health care resource use data collection.

Productivity losses will be assessed at baseline and at weeks 8 and 16 using the Work Productivity and Activity Impairment (WPAI) questionnaire [35], including the school version where applicable. Absenteeism, presenteeism, overall work or school impairment, and activity impairment will be derived using standard WPAI scoring algorithms. All participants will also report weekly hours spent managing their skin condition (eg, skin care routines, obtaining medications, and attending medical visits).

Weekly time losses, captured using a 7-day recall period, will be extrapolated over the relevant observation intervals and monetized using the national average hourly wage [36] to estimate time costs of disease management. Productivity losses associated with school absenteeism and impairment will be interpreted as near-term foregone or delayed paid-work productivity because all participants are aged 16 years or older, consistent with economic evaluation practice [37,38]. For the youth group, the national average wage for the 15- to 29-year age group will be applied [36].

Time spent using the MindMySkin app will be recorded but not costed, as engagement is expected to be brief, flexible, and unlikely to displace paid work or other productive activities, consistent with recommended practice for valuing time in digital intervention costing.

In Singapore, published cost-to-health care provider data are not available for most of the health care resources used. Hence, hospital charges are commonly used as proxies for economic costs in trial-based evaluations. As public hospitals operate on a cost-recovery basis with regulated pricing and minimal profit margins, patient charges (before subsidies) are considered reasonable approximations of the underlying cost of service from the health care system perspective.

### Measurement and Valuation of Health Outcomes

The primary health outcome for the cost-effectiveness analysis will be the DLQI, a disease-specific HRQOL scale ranging from 0 (no impairment) to 30 (maximum impairment). This is collected at baseline and at weeks 4, 8, 16, 24, and 32.

The EQ-5D-5L will be used for the cost-utility analysis, with utilities obtained from the Singapore value set for the measure [24,39]. Four skin-related bolt-ons will also be collected [40,41] and valued using available tariffs. The EQ-5D-5L data will be collected at baseline and at weeks 8, 16, and 32. The area under the curve for the 32-week study follow-up will be used to estimate the DLQI and QALYs gained over the study period. Differences between the comparator groups in QALY estimates will be assumed to result only from changes in HRQOL outcomes, with no change in life years.

### Statistical Analysis

#### Cost Analysis

Costs will be analyzed on an intention-to-treat basis. Means and SDs will be reported for all cost variables, even where distributions are skewed, with all observations retained in the primary analysis. Differences in mean costs between the intervention and control arms will be estimated using generalized linear models adjusting for baseline covariates. Mean costs for each arm and adjusted incremental cost differences will be reported with 95% CIs.

Where relevant, both subsidized and nonsubsidized cost structures will be considered, and the analytic perspective (health care system vs patient) will determine which values are used.

All costs will be expressed in Singapore dollars and adjusted to the price year of the final trial follow-up. Where cost data are obtained from earlier years, values will be inflated to the reference year using the Singapore Consumer Price Index for health care–related expenditures [42]. Discounting, whereby future costs and health outcomes are valued less than those occurring in the present, is unnecessary as the within-trial time horizon is less than 1 year.

#### Handling of Missing Data

Missing cost and outcome data will be handled using multiple imputation under the missing at random assumption. The imputation model will be stratified by the study arm and include demographic data, clinical data (disease type), and baseline health-related outcomes (utility value and HRQOL).

#### Cost-Effectiveness and Cost-Utility Analysis (Health Care Provider Perspective)

The base-case economic evaluation will be conducted from the health care system’s perspective and includes only direct medical costs. Economic outcomes will be summarized using incremental cost-effectiveness ratios (ICERs) and net monetary benefit (NMB).

ICERs, (C1–C0)/(Q1–Q0), will be estimated as the incremental cost per 1-point improvement in DLQI (cost-effectiveness analysis) and the incremental cost per QALY gained (cost-utility analysis) between the intervention and control arms. A regression model (eg, seemingly unrelated regression) will model the ICER, adjusting for variables such as age, gender, marital status, education, employment status, dermatological disease type, presence of psychiatric comorbidity at baseline, baseline utility values, and baseline HRQOL.

The NMB, representing how much treatment is worth in health terms, will be calculated as:


(1) NMB=(λ×ΔE)−ΔC

where *λ* (lambda) represents the *willingness-to-pay* (WTP) threshold, *ΔE* represents the incremental effect, and *ΔC* represents the incremental cost. As Singapore does not apply a single explicit cost-effectiveness threshold, results will be evaluated across a range of WTP values, in line with prior appraisals by the Agency for Care Effectiveness Singapore [43].

#### Cost-Effectiveness and Cost-Utility Analysis (Societal Perspective)

The societal perspective will incorporate out-of-pocket medical costs, nonmedical costs, and indirect costs associated with productivity losses in addition to direct medical costs. ICERs and NMBs will be estimated using the same process as that used for the health care provider perspective.

### Uncertainty and Sensitivity Analyses

Parameter, methodological, and structural uncertainty will be addressed through a series of prespecified sensitivity analyses conducted from the perspective of the primary health care system in various ways.

Sampling uncertainty in costs and outcomes will be characterized using nonparametric bootstrapping with 10,000 replications, resampling values with replacement. Bootstrap outputs will be used to derive 95% CIs for incremental costs, incremental effects, and ICERs. Uncertainty surrounding the ICERs will be illustrated using cost-effectiveness planes, while cost-effectiveness acceptability curves will be generated to depict the probability of the intervention being cost-effective across a range of WTP thresholds.

Parameter uncertainty will be explored through deterministic sensitivity analyses in which key cost inputs are varied within empirically derived bounds. To assess the influence of extreme cost observations, winsorization at the 95th percentile will be applied, whereby individual-level costs exceeding this threshold will be replaced with the 95th percentile value. Sensitivity analyses will also examine alternative assumptions regarding intervention costs and the projected user base to reflect uncertainty in implementation scale and market pricing.

Methodological uncertainty will be examined by estimating ICERs at an intermediate time point (week 16) in addition to the final follow-up at week 32, and by applying an alternative missing data strategy using complete-case analysis instead of multiple imputation.

Structural uncertainty will be assessed by testing alternative analytic assumptions, including the inclusion of intervention development costs under in-house development scenarios and the application of lower real-world effectiveness assumptions relative to trial conditions.

The impact of alternative assumptions regarding the potential user base and intervention uptake on fixed-cost allocation and per-user intervention costs will be explored.

### Heterogeneity and Subgroup Analyses

Heterogeneity will be assessed using a NMB regression framework, with interaction terms between treatment assignment and subgroup indicators to identify prognostic groups in which the intervention is cost-effective.

Adoption and engagement subgroups will be defined using app-derived use metrics, including overall module completion and the frequency and duration of app use. Demographic and socioeconomic subgroups will be examined by age group, education level, and employment status to assess distributional equity. Disease-related heterogeneity will be explored through primary dermatological diagnosis and baseline disease severity, including both clinician-assessed and patient-reported measures. Psychographic subgroups will examine baseline use of digital and mental health apps, resilience and self-efficacy, personality type, and the presence of comorbid anxiety or depression.

### Model-Based Extrapolation

If within-trial results suggest a high likelihood of cost-effectiveness over the trial follow-up, a model-based extrapolation will be undertaken to examine longer-term costs and outcomes over a 5-year horizon using a cohort Markov model with 6-month cycles. Health states will reflect clinically relevant dermatological trajectories, including controlled disease, disease flare, uncontrolled disease, and death. Transition probabilities, cost, and utility parameters will be informed by trial data where available and supplemented by local data, published literature, and expert input. Key assumptions will be explicitly stated and explored in sensitivity analyses, while model validity will be assessed through internal consistency checks and face validity. Where trial data are insufficient to reliably parameterize a Markov model, a simplified decision tree–based scenario analysis will be used.

### Ethical Considerations

Ethics approval for this study has been obtained from the National Healthcare Group Domain Specific Review Board (2022/00751). As the economic evaluation is conducted as a secondary analysis embedded within the parent RCT, no separate ethics application was required. Written informed consent is obtained from all participants prior to enrollment, including consent for collection of health care resource use and quality of life data for economic evaluation purposes. For minors, parental or guardian consent is obtained alongside participant assent, in accordance with institutional requirements. All data are deidentified prior to analysis, with participant information stored on password-protected institutional servers accessible only to authorized study personnel. Participants receive reimbursement of up to SG $80 (SG $1=US $ 0.77 as of June 29, 2026) upon completion of study assessments, disbursed incrementally across the different stages of the trial.

### Patient and Public Involvement

The study incorporates the insights and perspectives of patient and public representatives, from the development and curation of the psychotherapeutic modules to providing feedback on the study design. These members will also support the analysis and dissemination of the study results. There are currently 3 patient representatives involved in this study.

## Results

The study is funded by a National Medical Research Council Clinician-Scientist Individual Research Grant–New Investigator Grant (grant CNIG23jul-0003), awarded to the principal investigator (EC), covering the period from March 1, 2024, to February 28, 2027 (SG $259,740). Ethics approval was obtained from the National Healthcare Group Domain Specific Review Board (2022/00751), and the trial has been registered (ClinicalTrials.gov; NCT06702293). Site initiation and recruitment commenced at National University Hospital in February 2026, followed by National Skin Centre in March 2026 and KK Women’s and Children’s Hospital in April 2026.

As of June 2026, a total of 46 participants have been recruited across sites (National University Hospital: n=29, 56.5%; National Skin Centre: n=9, 35.8%; KK Women’s and Children’s Hospital: n=8, 8.7%). Recruitment is projected to reach the target sample of 690 participants by 2027. Economic data collection is proceeding concurrently with the parent RCT. No primary economic analyses have been conducted at this stage, as the protocol specifies that the primary economic analysis will commence upon trial completion. Publication of results is anticipated in 2028 to 2029. There have been no deviations from the registered protocol.

## Discussion

Digital mental health interventions are relatively inexpensive to scale and face fewer regulatory barriers than pharmaceuticals, contributing to their rapid expansion in the public market [44]. However, only a small proportion are developed through rigorous, theory-driven processes, and even fewer are evaluated in randomized trial settings [44], raising legitimate concerns regarding the safety and clinical effectiveness of the large majority of publicly available apps.

Additionally, while self-administered digital interventions are low in cost and easy to deploy, they generally produce smaller individual-level effects than therapist-delivered care. Hence, there remains a substantial evidence gap in cost-effectiveness, with recent systematic reviews published in 2025 identifying fewer than 30 digital health apps that underwent economic evaluation [45,46]. Furthermore, in an increasingly crowded digital marketplace, selective promotion and adoption are needed to avoid fragmentation and app fatigue. These considerations underscore the importance of assessing costs and outcomes in tandem to determine which interventions warrant institutional or national adoption.

Current economic evaluation frameworks are not well suited for digital and psychobehavioral interventions whose primary effects are functional rather than life-extending. Health systems have traditionally prioritized interventions that extend survival, potentially undervaluing psychosocial disability despite its substantial contribution to overall disease burden. For example, QALY estimates from the EQ-5D prioritize physical functioning domains and have been shown to underrepresent dermatological and psychosocial impacts [40,47]. In this study, we address these concerns by incorporating disease-specific measures and condition-relevant utility bolt-ons (skin-specific items appended to the EQ-5D), and in doing so, we hope to enhance sensitivity to disability-related change and align economic evaluation with what patients value most. Demonstrating economic value, even over a short-time horizon, would reinforce the legitimacy of low-intensity, scalable psychobehavioral interventions and contribute to the evolving international standards for digital mental health assessment.

Limitations include the use of self-reported health care use data for the estimation of costs due to the absence of comprehensive financial data or national benchmarks, which can lead to recall bias. This limitation is easily addressed in settings where such data are available by simply substituting the self-report estimates with actual values. The 32-week time horizon, while appropriate for capturing short- to medium-term differences attributable to the intervention, limits the assessment of longer-term costs and outcomes, which could be partially addressed through decision analytic modeling–based methods. Trial-based effectiveness estimates may also overestimate real-world effectiveness, as participant engagement and adherence in controlled trial settings typically exceed those observed under routine implementation conditions. Finally, restriction to English-speaking participants may introduce selection bias toward individuals who are more educated or of higher socioeconomic status, limiting the generalizability of findings to the broader population of patients with chronic inflammatory skin diseases in Singapore.

## Supplementary material

10.2196/95215 Checklist 1 CHEERS checklist.

10.2196/95215 Peer Review Report 1

10.2196/95215 Peer Review Report 2

## Abbreviations

DLQI: Dermatology Life Quality Index
HRQOL: health-related quality of life
ICER: incremental cost-effectiveness ratio
NMB: net monetary benefit
QALY: quality-adjusted life year
RCT: randomized controlled trial
WPAI: Work Productivity and Activity Impairment
WTP: willingness-to-pay
