# Supplementary material for: Economic Evaluation of a Digital Psychobehavioral Intervention for Chronic Inflammatory Skin Disease (MindMySkin): Protocol for a Trial-Based Cost-Effectiveness Analysis
Source: JMIR Res Protoc. 2026 Sep 29;15:e95215. doi: 10.2196/95215 (PMC13622875; doi:10.2196/95215)
Supplement: Supplementary file 1 [file resprot-v15-e95215-s002.pdf]

Summary of Panel Discussion

CNIG23jul-0003<br>Effectiveness of a Self-Administered Digital Psychological Toolkit to reducing Symptom Burden and Morbidity in Dermatology Patients: A Multicentre Randomised Controlled Trial<br>PI: Choi Ci En Ellie<br>HI: National University Hospital (NUH)<br>Amount: $259740<br>Duration: 36 months	- Lacked details on how the 8 point difference between the intervention arm and control arm was determined; lacked preliminary data to show the difference between the 2 arms<br>- Concerned about the feasibility of conducting a 750-patient study with the budget available<br>- Concerned about the lack of prior clinical trial experience in the PI and team<br>- Concerned on the study design; suggested to do an open label trial which might be more straightforward<br>- Questioned about the selection of applications as control; unsure if the available application would be sufficient as an effective control<br>- Mentor was based in US; concerned about the mentor's involvement and mentorship in the research<br>- Concerned about the grantsmanship<br>- Had potential to change clinical practice<br>- Lacked information on the primary outcome<br>- Concerned about the retention rate which might be an issue for such digital application study
