# Supplementary material for: Economic Evaluation of a Digital Psychobehavioral Intervention for Chronic Inflammatory Skin Disease (MindMySkin): Protocol for a Trial-Based Cost-Effectiveness Analysis
Source: JMIR Res Protoc. 2026 Sep 29;15:e95215. doi: 10.2196/95215 (PMC13622875; doi:10.2196/95215)
Supplement: Supplementary file 2 [file resprot-v15-e95215-s003.pdf]

Consolidated Review Report

**Programme name**	CNIG_R25
**Grant call name**	Clinician Scientist Individual Research Grant New Investigator Grant (CS-IRG-NIG) July 2023 Grant Call
**Reference ID**	CNIG23jul-0003
**Institution**	National University Hospital (NUH)
**Title of research project**	Effectiveness of a Self-Administered Digital Psychological Toolkit to reducing Symptom Burden and Morbidity in Dermatology Patients: A Multicentre Randomised Controlled Trial

PANEL REVIEWER 1 REPORT	
Question 1	**FEASIBILITY OF STUDY IN LOCAL CONTEXT - How feasible is the proposed approach? Is this likely to be the most effective way of tackling the problem? Has past work been exploited effectively, and/ or had significant impact on health care? Is the proposed work ethically acceptable? Are there any ethical issues that merit separate consideration? Are the funds requested essential for the work, and do the importance and scientific potential justify funding on the scale requested? Have the applicants clearly set out the reasons for requesting the level of staffing and overall resources? Is this level adequate for the work described? Taking into account the expected benefits of the work proposed and the level of resources requested, do the proposals promise good value for money?**
	Dermatological diseases result in significant distress and poor health. Quality of life of patients with chronic skin disease is often poor due to the associated psychological stress. This proposal aims to use scalable, digital based psychological interventions to improve mental health and quality of life of patients with dermatological diseases.<br>The PI has preliminary data to show that quality of life impairment correlated poorly with objective severity, but more strongly with anxiety and other psychological symptoms.<br>The PI has developed a prototype multimodal psychological intervention based on cognitive-behavioural constructs to improve symptom burden and quality of life<br>The methodology of the clinical trial is sound and the digital platform has several useful components including learning paths, knowledge delivery and psychological aids. The content will be delivered using a friendly interactive module.<br><br>The PI is working with a start-up in delivering the digital intervention. The digital app is meant to be fully automated and can be self-administered by patients. It is not entirely clear what is the role of the start-up. Is there IP sharing?<br>The patients in the control group will use the healthy365app, managed by HPB. The main outcome is a 8-week QoL. The 8-week duration seems rather short and may not be sufficiently long to manage psychological stress related to flare-up of the skin disease.<br>A 8-point change on the Skindex was used to calculate sample size. The PI fails to elaborate how this 8-point was determined.<br>The study aims to recruit 723 patients from multiple sits. I am uncertain if the budget allocated in a NIG of SGD 200k is sufficient for such a large trial. Will there be additional funding sources?
Question 2	**PRODUCTIVITY - Where relevant, are plans for organising research and fostering links to add value both realistic and likely to deliver higher quality or more productive science? Are there realistic approaches to the translation of research findings into improved practice in other fields and disciplines, whether more applied or fundamental?**
	There will be sites for this study with plans in place for effective translation of the findings.
Question 3	**TRACK RECORD OF INVESTIGATORS - Are the PIs, collaborators, and other researchers well suited to the project or have track record that is highly relevant to the proposed research? If they are in the early stages of independent careers, do they have appropriate experience and training? If established, have they demonstrated an ongoing record of accomplishments that have advanced their field(s)? If the project is collaborative or consists of multiple PIs, do the investigators have complementary and integrated expertise; are their leadership approach, governance and organizational structure appropriate for the project? Are the applicants uniquely placed to deliver the work? Is the productivity of the PI satisfactory in terms of the PI's achievements/progress in other NMRC grants?**
	The PI has published her work in high impact journals. The team has complimentary expertise.<br>It is not clear of the involvement of the industry start-up. Will they be supporting the project in any form? Will the IP be shared with the start-up?
Question 4	**OVERALL IMPACT ON HEALTH AND ECONOMIC OUTCOMES - Do the research project and its applications have the potential to advance human health and wellness and/or creating economic value for Singapore? Does the proposed project have the likelihood to exert sustained, powerful influence on the research field(s) involved? To what extent will the proposed translational research be able to significantly influence current or future medical practice and/or the cost-effectiveness of healthcare delivery?**
	The findings could make a big impact on the quality of life of patients with dermatological disease worldwide.

Question 5	**SUITABILITY OF APPLICANT - Comment on the suitability of the applicant to be an independent investigator and to assume the role of a Principal Investigator in the proposal.**
	The applicant has a good track record of research outcomes including high impact papers. The current project would allow her to further develop her research career.
Question 6	**RECOMMENDATIONS based on your assessment of the applicant's proposal.**
	Can be funded

PANEL REVIEWER 2 REPORT	
Question 1	**FEASIBILITY OF STUDY IN LOCAL CONTEXT - How feasible is the proposed approach? Is this likely to be the most effective way of tackling the problem? Has past work been exploited effectively, and/ or had significant impact on health care? Is the proposed work ethically acceptable? Are there any ethical issues that merit separate consideration? Are the funds requested essential for the work, and do the importance and scientific potential justify funding on the scale requested? Have the applicants clearly set out the reasons for requesting the level of staffing and overall resources? Is this level adequate for the work described? Taking into account the expected benefits of the work proposed and the level of resources requested, do the proposals promise good value for money?**
	Skin conditions would affect people's health and mind, and therefore their QOL. PI hypothesizes that a combination of psychobehavioral interventions through a mobile app can reduce the quality of life impairment in patients with chronic inflammatory skin disease. Application of digital technology has been gaining attention in the medical field, especially for expanding digital native population. This proposal is to perform multi-centre double blind randomized controlled clinical trial for digital app of psychobehavioral toolkit developed by them. This multimodal intervention contains 22 learning paths with 38 individual modules, 2 guided journals, 2 rescue sessions, and 1 weekly check-in.<br>It would be helpful to explain a bit more about this part, as generally psychobehavioral therapy has smaller effect size and therefore, what is unique about this toolkit, etc would be evaluated.<br>App to be used has already been developed. It would be nice to describe more about their app including accessibility, usability etc as this is a one of the key issues in digital technology application.<br>Control app to be used is always an issue in digital technology clinical trial. It is not clear why healthy 365 app is appropriate. Is that based on the usage frequency, activity, required brain activity etc? More description would be helpful.<br>How much they would have used app would be important in order to maximize the effect of the CBT. This is also applied for usage of the control app. In light of intended use of this for 4 months at least, this would be particularly important as the retention rate of the digital device is not that high for such a long time. It seems that usage of apps would be monitored (perhaps through like log in time duration and engagement monitoring etc). If so, personal information including usage would be treated as personal information and should be protected. There is no description on this part.<br>Funds are requested for one FTE for patient recruitment and for reimbursement for the participants. This sounds reasonable.
Question 2	**PRODUCTIVITY - Where relevant, are plans for organising research and fostering links to add value both realistic and likely to deliver higher quality or more productive science? Are there realistic approaches to the translation of research findings into improved practice in other fields and disciplines, whether more applied or fundamental?**
	Application of digital technology in medicine has been actively pursued all over the world. Issues are 1) evidence for the effectiveness for therapeutics and 2) implementation and market development for these new technology. For the former, effect size of the CBT has not been proven to be large. Retention rate of the usage of these kinds of digital technology has not been proven to be high if they intend that the patients would use them for several months. How to overcome these two points would require some consideration to be stated in the proposed study.
Question 3	**TRACK RECORD OF INVESTIGATORS - Are the PIs, collaborators, and other researchers well suited to the project or have track record that is highly relevant to the proposed research? If they are in the early stages of independent careers, do they have appropriate experience and training? If established, have they demonstrated an ongoing record of accomplishments that have advanced their field(s)? If the project is collaborative or consists of multiple PIs, do the investigators have complementary and integrated expertise; are their leadership approach, governance and organizational structure appropriate for the project? Are the applicants uniquely placed to deliver the work? Is the productivity of the PI satisfactory in terms of the PI's achievements/progress in other NMRC grants?**
	She has several Co-Is 1) who would help patient recruitment and clinical trial part, 2) who would help psychological tools and psychiatric followups, and 3) outcome choice, analysis and statistics. These would be sufficient to execute the proposed studies, assuming that they would not need to modify their app.<br>Would it be nice to have industrial personnel to be in the research group? especially if they would like to implement this in the future. It is stated that IP belongs to academia and is protected, but it is not clear how the industrial partner would be involved in this part. In addition, it is stated that the industrial partner is interested in implementing this platform, but it is not clear how that process would be materialized in light that they are not listed in the research group.

Question 4	**OVERALL IMPACT ON HEALTH AND ECONOMIC OUTCOMES - Do the research project and its applications have the potential to advance human health and wellness and/or creating economic value for Singapore? Does the proposed project have the likelihood to exert sustained, powerful influence on the research field(s) involved? To what extent will the proposed translational research be able to significantly influence current or future medical practice and/or the cost-effectiveness of healthcare delivery?**
	Applying digital technology in the medical field is expected to reduce the cost and work load of the work force. Effectiveness and access are the key for success. Assuming its effectiveness, some implementation plans for the apps would help to apply the developed app in clinical practice as digital therapeutics filed has an issue in creating appropriate and sustainable market, especially in medical personnel, i.e., medical doctors are busy in everyday work and would not use new technology unless they are absolutely needed to do.<br>It seems that PI has close connection with patient representatives/advocacy. That would help PI to be aligned with patients' needs and QOL in this project as well as in the future to improve the medical practice.
Question 5	**SUITABILITY OF APPLICANT - Comment on the suitability of the applicant to be an independent investigator and to assume the role of a Principal Investigator in the proposal.**
	PI is a dermatologist with some clinical research experience with several other trainings including business degree?. There is a mentor who is at Johns Hopkins Medical School in the US, but him nor any specific mentoring plan are not described in the proposal. It seems that she has been working independently and that she has collaborators in academia/clinical and in industry, who helped developing apps to be used in the proposal.
Question 6	**RECOMMENDATIONS based on your assessment of the applicant's proposal.**
	Original and innovative research project with potential for changing clinical practice. They already have app developed and this grant will support the clinical trial to test its effectiveness. It would be helpful to have colleagues who would help grant writing before submission.

PANEL REVIEWER 3 (READER*) REPORT

***Review comments from Reader are optional.**

Question 1	**FEASIBILITY OF STUDY IN LOCAL CONTEXT - How feasible is the proposed approach? Is this likely to be the most effective way of tackling the problem? Has past work been exploited effectively, and/ or had significant impact on health care? Is the proposed work ethically acceptable? Are there any ethical issues that merit separate consideration? Are the funds requested essential for the work, and do the importance and scientific potential justify funding on the scale requested? Have the applicants clearly set out the reasons for requesting the level of staffing and overall resources? Is this level adequate for the work described? Taking into account the expected benefits of the work proposed and the level of resources requested, do the proposals promise good value for money?**
	NA
Question 2	**PRODUCTIVITY - Where relevant, are plans for organising research and fostering links to add value both realistic and likely to deliver higher quality or more productive science? Are there realistic approaches to the translation of research findings into improved practice in other fields and disciplines, whether more applied or fundamental?**
	NA
Question 3	**TRACK RECORD OF INVESTIGATORS - Are the PIs, collaborators, and other researchers well suited to the project or have track record that is highly relevant to the proposed research? If they are in the early stages of independent careers, do they have appropriate experience and training? If established, have they demonstrated an ongoing record of accomplishments that have advanced their field(s)? If the project is collaborative or consists of multiple PIs, do the investigators have complementary and integrated expertise; are their leadership approach, governance and organizational structure appropriate for the project? Are the applicants uniquely placed to deliver the work? Is the productivity of the PI satisfactory in terms of the PI's achievements/progress in other NMRC grants?**
	NA
Question 4	**OVERALL IMPACT ON HEALTH AND ECONOMIC OUTCOMES - Do the research project and its applications have the potential to advance human health and wellness and/or creating economic value for Singapore? Does the proposed project have the likelihood to exert sustained, powerful influence on the research field(s) involved? To what extent will the proposed translational research be able to significantly influence current or future medical practice and/or the cost-effectiveness of healthcare delivery?**
	NA
Question 5	**SUITABILITY OF APPLICANT - Comment on the suitability of the applicant to be an independent investigator and to assume the role of a Principal Investigator in the proposal.**
	NA
Question 6	**RECOMMENDATIONS based on your assessment of the applicant's proposal.**
	NA
