# Supplementary material for: Economic Evaluation of a Digital Psychobehavioral Intervention for Chronic Inflammatory Skin Disease (MindMySkin): Protocol for a Trial-Based Cost-Effectiveness Analysis
Source: JMIR Res Protoc. 2026 Sep 29;15:e95215. doi: 10.2196/95215 (PMC13622875; doi:10.2196/95215)
Supplement: Supplementary file 3 [file resprot-v15-e95215-s001.docx]

# CHEERS 2022 Checklist

| **Section** | **No.** | **Guidance for Reporting** | **Reported in Manuscript** |
| --- | --- | --- | --- |
| **TITLE** | **1** | **Title**  *Identify the study as an economic evaluation and specify the interventions being compared.* | Title page, pg 1 |
| **ABSTRACT** | **2** | **Abstract**  *Provide a structured summary that highlights context, key methods, results and alternative analyses.* | Abstract section. pg 3 |
| **INTRODUCTION** | **3** | **Background and objectives**  *Give the context for the study, the study question and its practical relevance for decision making in policy or practice.* | Introduction section. pg 4 |
| **METHODS** | **4** | **Health economic analysis plan**  *Indicate whether a health economic analysis plan was developed and where available.* | Methods section (Economic Evaluation Framework). pg5 |
|  | **5** | **Study population**  *Describe characteristics of the study population (such as age range, demographics, socioeconomic, or clinical characteristics).* | Methods section (Study population and Setting). pg5 |
|  | **6** | **Setting and location**  *Provide relevant contextual information that may influence findings.* | Methods section (Study population and Setting; Study Perspective). pg5 |
|  | **7** | **Comparators**  *Describe the interventions or strategies being compared and why chosen.* | Methods section (Intervention and Comparator). pg5 |
|  | **8** | **Perspective**  *State the perspective(s) adopted by the study and why chosen.* | Methods section (Study Perspective). pg5 |
|  | **9** | **Time horizon**  *State the time horizon for the study and why appropriate.* | Methods section (Time Horizon). pg6 |
|  | **10** | **Discount rate**  *Report the discount rate(s) and reason chosen.* | Methods section (Time Horizon). Discounting not applicable as the within-trial time horizon is less than one year. pg6 |
|  | **11** | **Selection of outcomes**  *Describe what outcomes were used as the measure(s) of benefit(s) and harm(s).* | Methods section (Measurement and Valuation of Health Outcomes). pg10-11 |
|  | **12** | **Measurement of outcomes**  *Describe how outcomes used to capture benefit(s) and harm(s) were measured.* | Methods section (Measurement and Valuation of Health Outcomes). pg10-11 |
|  | **13** | **Valuation of outcomes**  *Describe the population and methods used to measure and value outcomes.* | Methods section (Measurement and Valuation of Health Outcomes). pg10-11 |
|  | **14** | **Measurement and valuation of resources and costs**  *Describe how costs were valued.* | Methods section (Measurement and Valuation of Costs) pg6 |
|  | **15** | **Currency, price date, and conversion**  *Report the dates of the estimated resource quantities and unit costs, plus the currency and year of conversion.* | Methods section (Cost Analysis). pg6 |
|  | **16** | **Rationale and description of model**  *If modelling is used, describe in detail and why used.* | Methods section (Model-Based Extrapolation). pg13 |
|  | **17** | **Analytics and assumptions**  *Describe any methods for analysing or statistically transforming data, any extrapolation methods, and approaches for validating any model used.* | Methods section (Statistical Analysis; Cost-effectiveness Analysis). pg11 |
|  | **18** | **Characterizing heterogeneity**  *Describe any methods used for estimating how the results of the study vary for sub-groups.* | Methods section (Heterogeneity and Subgroup Analyses). pg12 |
|  | **19** | **Characterizing distributional effects**  *Describe how impacts are distributed across different individuals or adjustments made to reflect priority populations.* | Methods section (Heterogeneity and Subgroup Analyses) pg12 |
|  | **20** | **Characterizing uncertainty**  *Describe methods to characterize any sources of uncertainty in the analysis.* | Methods section (Uncertainty and Sensitivity Analyses). pg12 |
|  | **21** | **Approach to engagement with patients and others affected by the study**  *Describe any approaches to engage patients or service recipients, the general public, communities, or stakeholders in the design of the study.* | Methods section (Patient and public involvement). pg14 |
| **RESULTS** | **22** | **Study parameters**  *Report all analytic inputs (e.g., values, ranges, references) including uncertainty or distributional assumptions.* | NA (nil results yet) |
|  | **23** | **Summary of main results**  *Report the mean values for the main categories of costs and outcomes of interest and summarise them in the most appropriate overall measure.* | NA (nil results yet) |
|  | **24** | **Effect of uncertainty**  *Describe how uncertainty about analytic judgments, inputs, or projections affect findings.* | NA (nil results yet) |
|  | **25** | **Effect of engagement with patients and others affected by the study**  *Report on any difference patient/service recipient, general public, community, or stakeholder involvement made to the approach or findings of the study.* | NA (nil results yet) |
| **DISCUSSION** | **26** | **Study findings, limitations, generalizability, and current knowledge**  *Report key findings, limitations, ethical or equity considerations not captured, and how these could impact patients, policy or practice.* | Discussion section |
| **OTHER** | **27** | **Source of funding**  *Describe how the study was funded and any role of the funder in the identification, design, conduct, and reporting of the analysis.* | Funding statement. pg2 |
|  | **28** | **Conflicts of interest**  *Report authors conflicts of interest according to journal or ICMJE requirements.* | Competing interests statement. pg2 |
